# (*E*)-3-Dimethyl­amino-1-(2-pyrid­yl)prop-2-en-1-one

**DOI:** 10.1107/S1600536809030530

**Published:** 2009-08-08

**Authors:** Lei Ni, Ji-Li Zhao, Hong Wei

**Affiliations:** a College of Chemistry and Biology, Beihua University, Jilin 132013, People’s Republic of China

## Abstract

The mol­ecule of the title compound, C_10_H_12_N_2_O, is approximately planar, with an r.m.s. deviation of 0.072 Å from the mean plane for the non-H atoms. It was synthesized from 2-acetyl­pyridine and *N*,*N*-dimethyl­formamide dimethyl acetal in a one-step reaction.

## Related literature

For background to related heteroaromatic compounds, see: Zhang *et al.* (2009 ▶); Liu *et al.* (2009 ▶); Kida *et al.* (2009 ▶).
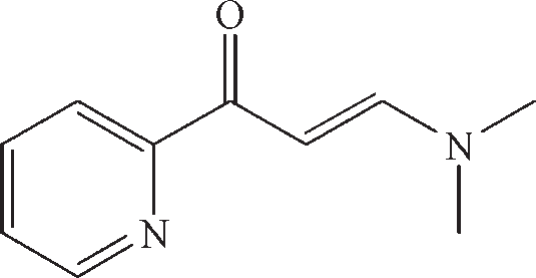


## Experimental

### 

#### Crystal data

C_10_H_12_N_2_O
*M* *_r_* = 176.22
Monoclinic, 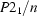

*a* = 5.6670 (11) Å
*b* = 23.117 (5) Å
*c* = 7.6880 (15) Å
β = 108.17 (3)°
*V* = 956.9 (3) Å^3^
*Z* = 4
Mo *K*α radiation
μ = 0.08 mm^−1^
*T* = 295 K
0.12 × 0.10 × 0.08 mm

#### Data collection

Bruker APEXII CCD diffractometer
Absorption correction: multi-scan (*SADABS*; Bruker, 2004 ▶) *T*_min_ = 0.990, *T*_max_ = 0.994
7131 measured reflections
1775 independent reflections
1403 reflections with *I* > 2σ(*I*)
*R*_int_ = 0.027

#### Refinement

*R*[*F* ^2^ > 2σ(*F* ^2^)] = 0.047
*wR*(*F* ^2^) = 0.143
*S* = 1.00
1775 reflections
121 parameters
H-atom parameters not refined
Δρ_max_ = 0.18 e Å^−3^
Δρ_min_ = −0.15 e Å^−3^

### 

Data collection: *APEX2* (Bruker, 2004 ▶); cell refinement: *SAINT-Plus* (Bruker, 2004 ▶); data reduction: *SAINT-Plus*; program(s) used to solve structure: *SHELXS97* (Sheldrick, 2008 ▶); program(s) used to refine structure: *SHELXL97* (Sheldrick, 2008 ▶); molecular graphics: *SHELXTL* (Sheldrick, 2008 ▶); software used to prepare material for publication: *SHELXTL*.

## Supplementary Material

Crystal structure: contains datablocks I, global. DOI: 10.1107/S1600536809030530/hb5023sup1.cif

Structure factors: contains datablocks I. DOI: 10.1107/S1600536809030530/hb5023Isup2.hkl

Additional supplementary materials:  crystallographic information; 3D view; checkCIF report

## Figures and Tables

**Fig. 1.**
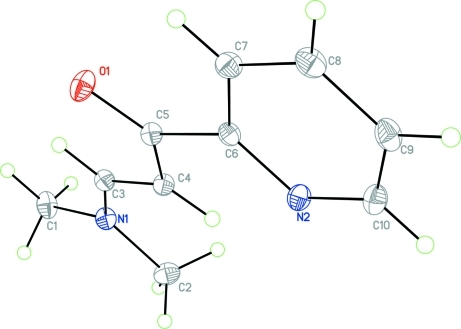
The molecular structure of (I), drawn with 30% probability displacement ellipsoids for the non-hydrogen atoms.

## supplementary crystallographic information

### Comment

Derives of heteroaromatic ligands have proven to be extremely popular amongst coordination chemists for a wide range of applications because of their ease of synthesis, ease of functionalization, and the steric protection which they afford to metal centres (Zhang *et al.*; Liu *et al.*; Kida *et al.*). Here, we report the synthesis and structure of the title compound, (I).

As shown in Fig. 1, non-hydrogen atoms including the pyridine ring, *N*,*N*-Dimethylamino, and prop-2-en-1-one are coplanar with Rms deviation of fitted atoms being 0.0724 Å. The title compound is synthesized from 2-acetylpyridine and *N*,*N*-dimethylformamide-dimethyl acetal by one step.

### Experimental

A mixture of 2-acetylpyridine(10 mmol) and *N*,*N*-dimethylformamide-dimethyl acetal(40 ml) was refluxed for four hours. After concentration in vacuo, recrystallization of the orange residue from ethanol afforded yellow blocks of (I). Anal. Calc. for C_10_H_12_N_2_O: C 68.10, H 6.81, N 15.89%; Found: C 68.02, H 6.63, N 15.79%.

### Refinement

All H atoms were geometrically positioned (C—H = 0.93–0.97Å) and refined as riding with *U*_iso_(H) = 1.2U_eq_(C) or 1.5U_eq_(methyl C).

### Crystal data

**Table:**

C_10_H_12_N_2_O	*F*(000) = 376
*M**_r_* = 176.22	*D*_x_ = 1.223 Mg m^−^^3^
Monoclinic, *P*2_1_/*n*	Mo *K*α radiation, λ = 0.71073 Å
Hall symbol: -P 2yn	Cell parameters from 1775 reflections
*a* = 5.6670 (11) Å	θ = 1.8–25.5°
*b* = 23.117 (5) Å	µ = 0.08 mm^−^^1^
*c* = 7.6880 (15) Å	*T* = 295 K
β = 108.17 (3)°	Block, yellow
*V* = 956.9 (3) Å^3^	0.12 × 0.10 × 0.08 mm
*Z* = 4	

### Data collection

**Table:**

Bruker APEXII CCD diffractometer	1775 independent reflections
Radiation source: fine-focus sealed tube	1403 reflections with *I* > 2σ(*I*)
graphite	*R*_int_ = 0.027
φ and ω scans	θ_max_ = 25.5°, θ_min_ = 1.8°
Absorption correction: multi-scan (*SADABS*; Bruker, 2004)	*h* = −6→6
*T*_min_ = 0.990, *T*_max_ = 0.994	*k* = −27→28
7131 measured reflections	*l* = −9→9

### Refinement

**Table:**

Refinement on *F*^2^	Primary atom site location: structure-invariant direct methods
Least-squares matrix: full	Secondary atom site location: difference Fourier map
*R*[*F*^2^ > 2σ(*F*^2^)] = 0.047	Hydrogen site location: inferred from neighbouring sites
*wR*(*F*^2^) = 0.143	H-atom parameters not refined
*S* = 1.00	*w* = 1/[σ^2^(*F*_o_^2^) + (0.087*P*)^2^ + 0.1213*P*] where *P* = (*F*_o_^2^ + 2*F*_c_^2^)/3
1775 reflections	(Δ/σ)_max_ < 0.001
121 parameters	Δρ_max_ = 0.18 e Å^−^^3^
0 restraints	Δρ_min_ = −0.15 e Å^−^^3^

### Special details

**Table:**

Geometry. All e.s.d.'s (except the e.s.d. in the dihedral angle between two l.s. planes) are estimated using the full covariance matrix. The cell e.s.d.'s are taken into account individually in the estimation of e.s.d.'s in distances, angles and torsion angles; correlations between e.s.d.'s in cell parameters are only used when they are defined by crystal symmetry. An approximate (isotropic) treatment of cell e.s.d.'s is used for estimating e.s.d.'s involving l.s. planes.
Refinement. Refinement of *F*^2^ against ALL reflections. The weighted *R*-factor *wR* and goodness of fit *S* are based on *F*^2^, conventional *R*-factors *R* are based on *F*, with *F* set to zero for negative *F*^2^. The threshold expression of *F*^2^ > σ(*F*^2^) is used only for calculating *R*-factors(gt) *etc*. and is not relevant to the choice of reflections for refinement. *R*-factors based on *F*^2^ are statistically about twice as large as those based on *F*, and *R*- factors based on ALL data will be even larger.

### Fractional atomic coordinates and isotropic or equivalent isotropic displacement parameters (Å^2^)

**Table:**

	*x*	*y*	*z*	*U*_iso_*/*U*_eq_	
C1	0.3675 (4)	0.01987 (8)	−0.3188 (3)	0.0664 (6)	
H1A	0.2562	−0.0080	−0.2945	0.100*	
H1B	0.5232	0.0016	−0.3092	0.100*	
H1C	0.2964	0.0351	−0.4401	0.100*	
C2	0.5845 (4)	0.11075 (9)	−0.1969 (3)	0.0674 (6)	
H2A	0.5072	0.1481	−0.2079	0.101*	
H2B	0.6385	0.1038	−0.3017	0.101*	
H2C	0.7251	0.1096	−0.0879	0.101*	
C3	0.2893 (3)	0.06861 (7)	−0.0630 (2)	0.0456 (4)	
H3	0.1843	0.0377	−0.0633	0.055*	
C4	0.3024 (3)	0.11046 (7)	0.0648 (2)	0.0464 (4)	
H4	0.4006	0.1430	0.0703	0.056*	
C5	0.1648 (3)	0.10380 (7)	0.1890 (2)	0.0445 (4)	
C6	0.1719 (3)	0.15207 (6)	0.3237 (2)	0.0423 (4)	
C7	0.0089 (3)	0.15063 (8)	0.4258 (2)	0.0533 (5)	
H7	−0.1038	0.1204	0.4118	0.064*	
C8	0.0165 (4)	0.19467 (9)	0.5484 (2)	0.0615 (5)	
H8	−0.0916	0.1947	0.6179	0.074*	
C9	0.1851 (4)	0.23816 (8)	0.5661 (2)	0.0608 (5)	
H9	0.1951	0.2682	0.6484	0.073*	
C10	0.3406 (4)	0.23655 (8)	0.4592 (2)	0.0584 (5)	
H10	0.4549	0.2664	0.4720	0.070*	
N1	0.4076 (3)	0.06659 (6)	−0.18682 (18)	0.0500 (4)	
N2	0.3368 (3)	0.19482 (6)	0.33867 (18)	0.0505 (4)	
O1	0.0370 (3)	0.06092 (5)	0.19576 (18)	0.0657 (4)	

### Atomic displacement parameters (Å^2^)

**Table:**

	*U*^11^	*U*^22^	*U*^33^	*U*^12^	*U*^13^	*U*^23^
C1	0.0977 (16)	0.0551 (11)	0.0587 (11)	0.0089 (10)	0.0423 (11)	−0.0012 (9)
C2	0.0611 (12)	0.0857 (14)	0.0628 (12)	−0.0074 (10)	0.0303 (10)	0.0026 (10)
C3	0.0508 (9)	0.0462 (9)	0.0432 (9)	0.0036 (7)	0.0197 (7)	0.0052 (7)
C4	0.0487 (9)	0.0464 (9)	0.0466 (9)	−0.0007 (7)	0.0185 (7)	−0.0010 (7)
C5	0.0491 (9)	0.0431 (9)	0.0427 (9)	0.0008 (7)	0.0164 (7)	0.0007 (7)
C6	0.0457 (9)	0.0423 (9)	0.0387 (8)	0.0060 (7)	0.0128 (7)	0.0034 (6)
C7	0.0541 (10)	0.0571 (11)	0.0531 (10)	0.0016 (8)	0.0232 (8)	−0.0037 (8)
C8	0.0649 (12)	0.0701 (13)	0.0560 (11)	0.0110 (10)	0.0284 (9)	−0.0077 (9)
C9	0.0763 (13)	0.0555 (11)	0.0482 (10)	0.0122 (9)	0.0161 (9)	−0.0103 (8)
C10	0.0730 (12)	0.0476 (10)	0.0519 (10)	−0.0048 (9)	0.0158 (9)	−0.0051 (8)
N1	0.0591 (9)	0.0520 (8)	0.0462 (8)	0.0031 (6)	0.0271 (7)	0.0023 (6)
N2	0.0581 (9)	0.0476 (8)	0.0466 (8)	−0.0037 (7)	0.0178 (7)	−0.0031 (6)
O1	0.0896 (10)	0.0554 (8)	0.0676 (9)	−0.0208 (7)	0.0472 (8)	−0.0144 (6)

### Geometric parameters (Å, °)

**Table:**

C1—N1	1.451 (2)	C5—O1	1.2384 (18)
C1—H1A	0.9600	C5—C6	1.514 (2)
C1—H1B	0.9600	C6—N2	1.340 (2)
C1—H1C	0.9600	C6—C7	1.386 (2)
C2—N1	1.450 (2)	C7—C8	1.379 (2)
C2—H2A	0.9600	C7—H7	0.9300
C2—H2B	0.9600	C8—C9	1.364 (3)
C2—H2C	0.9600	C8—H8	0.9300
C3—N1	1.325 (2)	C9—C10	1.380 (3)
C3—C4	1.364 (2)	C9—H9	0.9300
C3—H3	0.9300	C10—N2	1.333 (2)
C4—C5	1.417 (2)	C10—H10	0.9300
C4—H4	0.9300		
			
N1—C1—H1A	109.5	C4—C5—C6	118.57 (14)
N1—C1—H1B	109.5	N2—C6—C7	122.61 (15)
H1A—C1—H1B	109.5	N2—C6—C5	118.10 (14)
N1—C1—H1C	109.5	C7—C6—C5	119.29 (15)
H1A—C1—H1C	109.5	C8—C7—C6	119.01 (17)
H1B—C1—H1C	109.5	C8—C7—H7	120.5
N1—C2—H2A	109.5	C6—C7—H7	120.5
N1—C2—H2B	109.5	C9—C8—C7	118.94 (17)
H2A—C2—H2B	109.5	C9—C8—H8	120.5
N1—C2—H2C	109.5	C7—C8—H8	120.5
H2A—C2—H2C	109.5	C8—C9—C10	118.55 (16)
H2B—C2—H2C	109.5	C8—C9—H9	120.7
N1—C3—C4	128.04 (15)	C10—C9—H9	120.7
N1—C3—H3	116.0	N2—C10—C9	123.96 (17)
C4—C3—H3	116.0	N2—C10—H10	118.0
C3—C4—C5	119.39 (15)	C9—C10—H10	118.0
C3—C4—H4	120.3	C3—N1—C2	121.81 (14)
C5—C4—H4	120.3	C3—N1—C1	121.65 (15)
O1—C5—C4	124.55 (15)	C2—N1—C1	116.54 (14)
O1—C5—C6	116.87 (14)	C10—N2—C6	116.93 (15)
